# Systemic and heart autonomous effects of sphingosine Δ4 desaturase deficiency in lipotoxic cardiac pathophysiology

**DOI:** 10.1242/dmm.043083

**Published:** 2020-08-14

**Authors:** Stanley M. Walls, Dale A. Chatfield, Karen Ocorr, Greg L. Harris, Rolf Bodmer

**Affiliations:** 1 Development, Aging and Regeneration Program, Sanford-Burnham-Prebys Medical Discovery Institute, La Jolla, CA 92037, USA; 2 Department of Cellular and Molecular Biology, San Diego State University, San Diego, CA 92182, USA

**Keywords:** Ifc, Sphingosine Δ4 desaturase, Dihydroceramide, Sphingolipid metabolism, Ceramide, Cardiac

## Abstract

Lipotoxic cardiomyopathy (LCM) is characterized by cardiac steatosis, including the accumulation of fatty acids, triglycerides and ceramides. Model systems have shown the inhibition of ceramide biosynthesis to antagonize obesity and improve insulin sensitivity. Sphingosine Δ4 desaturase (encoded by *ifc* in *Drosophila melanogaster*) enzymatically converts dihydroceramide into ceramide. Here, we examine *ifc* mutants to study the effects of desaturase deficiency on cardiac function in *Drosophila*. Interestingly, *ifc* mutants exhibited classic hallmarks of LCM: cardiac chamber dilation, contractile defects and loss of fractional shortening. This outcome was phenocopied in global *ifc* RNAi-mediated knockdown flies. Surprisingly, cardiac-specific *ifc* knockdown flies exhibited cardiac chamber restriction with no contractile defects, suggesting heart autonomous and systemic roles for *ifc* activity in cardiac function. Next, we demonstrated that *ifc* mutants exhibit suppressed *Sphingosine kinase 1* (*Sk1*) expression. Ectopic overexpression of *Sk1* was sufficient to prevent cardiac chamber dilation and loss of fractional shortening in *ifc* mutants. Partial rescue was also observed with cardiac- and fat-body-specific *Sk1* overexpression. Finally, we showed that cardiac-specific expression of *Drosophila* inhibitor of apoptosis (dIAP) also prevented cardiac dysfunction in *ifc* mutants, suggesting a role for caspase activity in the observed cardiac pathology. Collectively, we show that spatial regulation of sphingosine Δ4 desaturase activity differentially affects cardiac function in heart autonomous and systemic mechanisms through tissue interplay.

## Abstract (precis)

**Summary:** Systemic versus heart autonomous functions of sphingosine Δ4 desaturase differentially regulate cardiac structure and function in a tissue-specific manner in *Drosophila*, where organ interplay mimics that observed in mammalian systems.

## INTRODUCTION

Sphingolipid metabolism is an important cellular pathway for the synthesis of key regulatory bioactive lipids including ceramide and sphingosine 1-phosphate. Sphingolipid intermediates and metabolites, beyond their structural role in membranes, can have a variety of molecular roles in cellular processes, including autophagy (Harvald et al., 2015), cell signaling (Smith and Merrill, 2002), apoptosis and proliferation (Mullen and Obeid, 2012). In recent years, myocardial sphingolipid metabolism has been shown to have a role in the pathogenesis of cardiac disease. Although ceramide, the metabolic hub of sphingolipid metabolism, has been shown to exhibit cardiotoxic properties in the progression of lipotoxic (obesity and diabetic) cardiomyopathies (Bandet et al., 2019; Park et al., 2008; Walls et al., 2018), sphingosine 1-phosphate has been shown to be cardioprotective (Knapp, 2011; Ahmed et al., 2019).

However, less is known regarding the role of other sphingolipid intermediates, including dihydroceramide, in the etiology of these diseases. In *Drosophila*, the gene *infertile crescent* (*ifc*) encodes for the protein sphingosine Δ4 desaturase, which catalyzes the introduction of a double bond between C4 and C5 on the sphingoid backbone of dihydroceramide, the final step in *de novo* ceramide synthesis (Ternes et al., 2002). Sphingosine Δ4 desaturase has been implicated in a diverse set of biological processes, including photoreceptor cell maintenance (Huang et al., 2015), spermatogenesis (Castrillon et al., 1993) and spindle assembly in male meiosis (Basu and Li, 1998). Mutant female *ifc^4^* flies also exhibit a myriad of reproductive defects, including malformed oocytes, follicle degeneration, egg retention and supernumerary spermathecae (Phan et al., 2007). However, the regulatory role of this protein in the heart is not completely understood.

More recent studies have shown that *ifc^4^* mutants also exhibit classic hallmarks of obesity, including elevated triglyceride (TG) levels, fat body (adipocyte) hypertrophy and resistance to starvation-induced expiration (Walls et al., 2013). These studies suggest that the lipid accumulation reported in *ifc^4^* mutants was directly associated with the loss of adipokinetic hormone (Akh)-mediated fat mobilization. Akh in *Drosophila* works as a functional ortholog, like β-adrenergic agonists or glucagon, and promotes the breakdown of glycogen and TGs for energy utilization (Gronke et al., 2007). Thus, *ifc*-encoded sphingosine Δ4 desaturase activity appears to be necessary for Akh-producing cell (Akhpc) viability and function in flies.

These findings correlate with previous studies which showed that apoptosis-induced cell death of Akhpc suppressed TG mobilization from *Drosophila* fat-body cells (Gronke et al., 2007). Similarly, Akhpc-specific knockdown of *ifc* led to fewer Akhpc in 3rd instar larvae relative to control. By adulthood, Akhpc and *akh* transcripts were nearly undetectable in Akhpc-*ifc* RNA interference (RNAi) knockdown flies (Walls et al., 2013). Predictably, these flies also exhibited increased TG levels, which could be alleviated by concomitant Akhpc-specific overexpression of *Drosophila* *inhibitor of apoptosis* (*dIAP*; also known as *Diap1*).

Thus, sphingosine Δ4 desaturase deficiency induced lipotoxicity and cell death in Akhpc, leading to a loss of Akh-mediated TG mobilization, accumulation of lipotoxic sphingolipid intermediates and an obese phenotype (Walls et al., 2013). Given the established associations between obesity and heart disease, we sought to characterize the role of sphingosine Δ4 desaturase in cardiac function.

In this study, we found that *ifc^4^* mutants, which accumulate excess TG and an aberrant sphingolipid profile (Walls et al., 2013), develop severe heart dysfunction including diastolic and systolic cardiac dilation and loss of fractional shortening. Similar cardiac phenotypes are also observed in flies with global RNAi-mediated knockdown of *ifc* mRNA. Unexpectedly, when we knockdown *ifc* specifically in the heart, the hearts of these flies are not dilated but instead exhibit diastolic and systolic cardiac chamber restriction. The restricted cardiac phenotype is reminiscent of serine palmitoyl transferase (*lace*) and ceramide synthase (*schlank*) global and heart-specific knockdowns (Walls et al., 2018), suggesting that this cardiac restriction phenotype might be due to genetic suppression of *de novo* ceramide synthesis.

To gain further insight into the cause of the disparate phenotypes exhibited between global versus cardiac-specific knockdown of *ifc*, we examined the expression levels of other sphingolipid metabolic genes in *ifc* mutants. Indeed, *ifc^4^* mutant flies exhibit decreased global expression of *Sphingosine kinase 1* (*Sk1*), which appears to account, in part, for the differential phenotype exhibited by global versus cardiac-specific *ifc* knockdown flies, as *Sk1* is not expressed in the heart. Specifically, we show that global overexpression of *Sk1* in *ifc^4^* mutants prevents cardiac dilation and normalizes contractility. Next, we show that overexpression of *Sk1* specifically in the heart or fat body partially rescues the *ifc^4^* mutant cardiac phenotypes, suggesting that cardiac function might be regulated by sphingolipid metabolite modulation both systemically and in the heart. Restoration of Akhpcs, through the Akhpc-specific overexpression of *dIAP*, also partially restored *ifc^4^* mutant cardiac function, presumably by reactivation of TG utilization in the heart and periphery. Finally, cardiac-specific overexpression of *dIAP* was sufficient to rescue the *ifc^4^* mutant phenotype, suggesting that the cardiac phenotype is at least partially caspase dependent. These results correlate strongly with the previously reported cardiac-specific *dIAP* overexpression rescue of ceramide dietary-induced cardiac dilation and contractile dysfunction (Walls et al., 2018).

## RESULTS

### Sphingosine Δ4 desaturase *ifc^4^* mutants exhibit cardiac defects

**Fig. 1.**
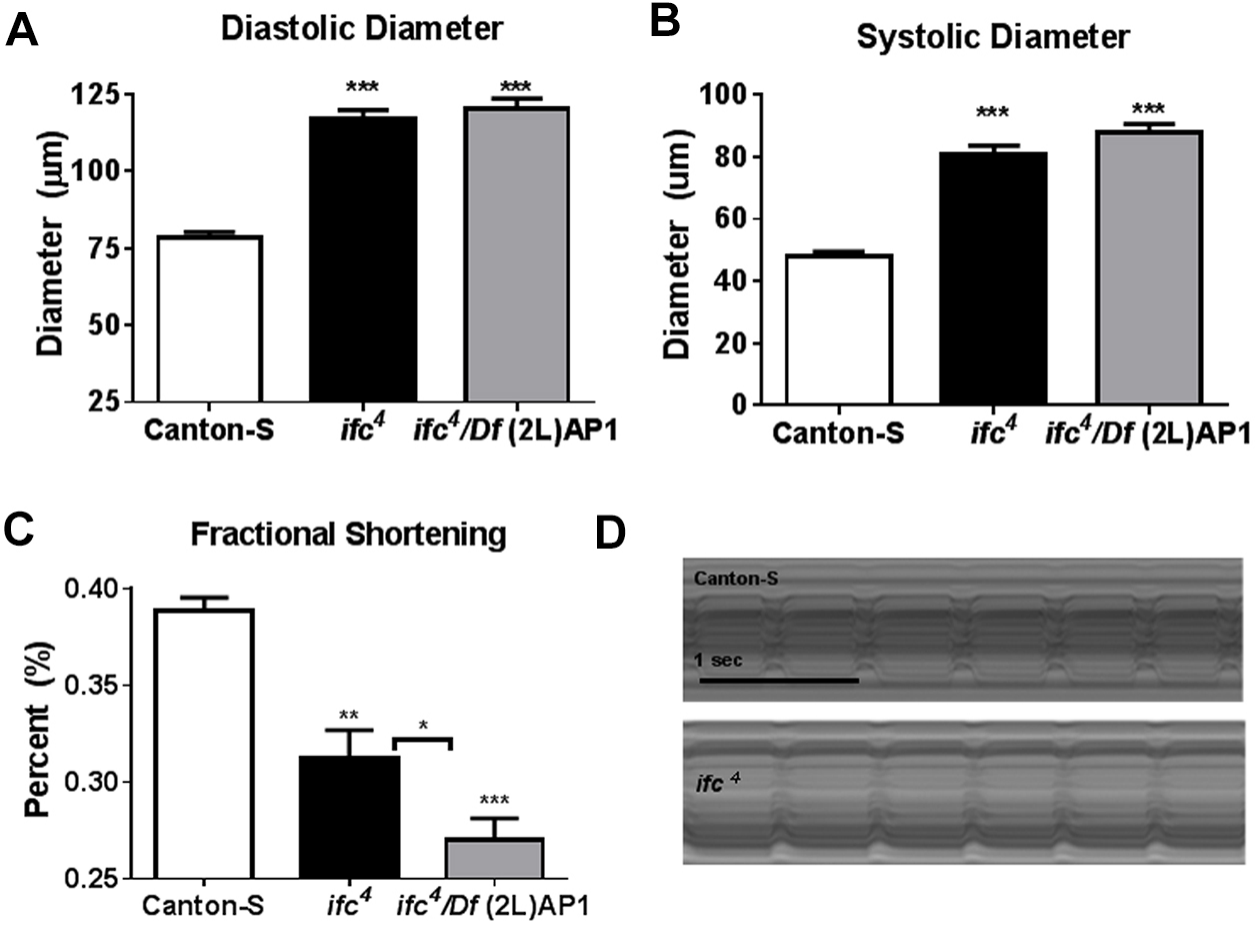
**Mutant *ifc^4^* flies exhibit dilated cardiac dysfunction.** (A-D) Both *ifc^4^* mutants and *ifc^4^*/*Df(2L) AP1* transheterozygotes exhibit an increase in diastolic diameter (A) and systolic diameter (B), and a reduction in fractional shortening (C), relative to Canton-S (CS) wild-type controls, as observed in representative M-modes (D). Pairwise two-tailed Student's *t*-test. *P<0.05; **P<0.01; ***P<0.001. Error bars=s.e.m. *n*=30 flies.

Mutant *ifc^4^* flies have been reported to exhibit obese phenotypes with the accumulation of TG and sphingolipid subspecies, including dihydroceramide, dihydrosphingosine and ceramide/sphingosine dienes, which correlated with a global loss of *ifc* mRNA expression (Walls et al., 2013). To determine the effects of this shift in lipid profile on cardiac function, we employed high-speed video microscopy on semi-intact 3-week-old female *ifc^4^* fly heart preparations (Fink et al., 2009; Ocorr et al., 2009). Relative to wild-type flies, *ifc^4^* mutants exhibited dilated diastolic and systolic diameters (Fig. 1A,B) with a concomitant loss in fractional shortening (Fig. 1C). However, no changes in heart beat length (heart period) or rhythmicity were observed, as illustrated by a representative M-mode (Fig. 1D). Similar phenotypes were observed in *ifc^4^ in trans* to a deficiency of the locus [*Df(2L)AP1*], thus substantiating the hypothesis that it is indeed the loss of *ifc* gene function that causes severe cardiac dilation and compromised contractility. This finding correlated with an observed loss of *ifc* expression in the hearts of *ifc^4^* mutants (Fig. 1). Taken together, these data suggest that sphingosine Δ4 desaturase mutants exhibit a lipotoxic cardiomyopathy-like phenotype.

### Systemic versus heart-specific knockdown of *ifc* differentially affects cardiac function

**Fig. 2.**
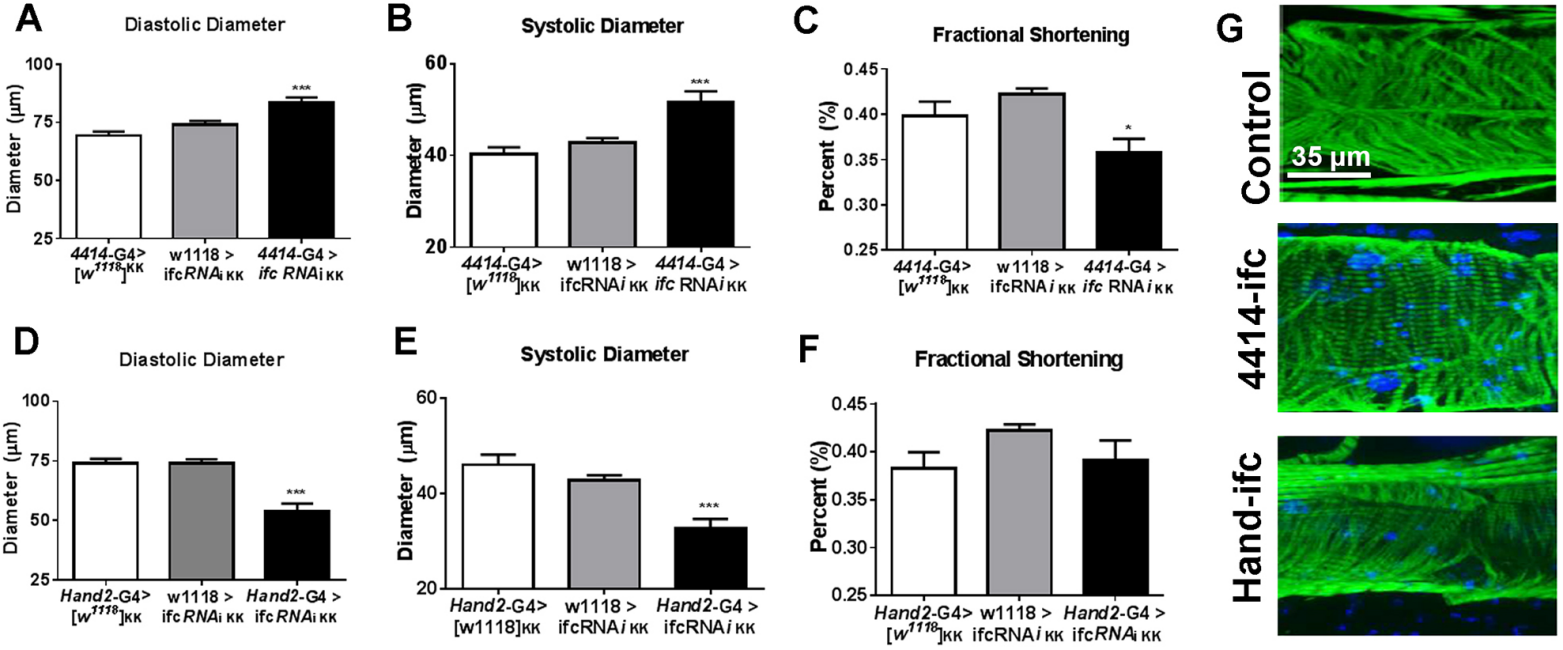
**Spatial control of *ifc* KD confers unique cardiac phenotypes.** (A-C) Global RNAi-mediated KD of *ifc* (4414-G4>*ifc* RNAi) induces similar phenotypes to those observed in *ifc^4^* mutants, including increased diastolic diameters (A) and increased systolic diameters (B), with a loss of fractional shortening (C), relative to driver control [4414 (actin)-G4>*w^1118^*] and UAS control (*w^1118^*>*ifc* RNAi) flies. (D-F) Conversely, cardiac-specific KD of *ifc* (Hand2-G4>*ifc* RNAi) induces a restricted phenotype, including a reduction in diastolic diameter (D) and systolic diameter (E), with no change in fractional shortening (F). (G) Representative cardiac tubes are shown: green, phalloidin F-actin stain; blue, DAPI nuclear stain (not used in driver controls). Multiple comparisons two-tailed Student's *t*-test. *P<0.05; ***P<0.001. Error bars=s.e.m. *n*=30 flies.

To determine whether the observed cardiac defects occurred specifically from loss of sphingosine Δ4 desaturase function in the heart or through the systemic effects of sphingosine Δ4 desaturase loss, we utilized a Gal4/UAS RNAi-mediated knockdown (KD) approach (Brand and Perrimon, 1993). As in *ifc^4^* mutants, flies with global KD of *ifc* using an actin-Gal4 driver exhibit significant loss of *ifc* mRNA expression (Walls et al., 2013). Global *ifc* KD fly hearts also showed increased diastolic and systolic diameters (Fig. 2A,B). A significant loss in fractional shortening was also observed.

Next, we examined the effects of heart-specific KD of *ifc* on cardiac function using the Hand-Gal4 driver (Han and Olson, 2005). Strikingly, flies with heart-specific KD of *ifc* exhibited constricted rather than dilated cardiac chamber width, with a reduction in both diastolic and systolic diameter, but with no significant change in fractional shortening (Fig. 2D-F). Based on myofibrillar staining, heart-specific *ifc* KD hearts also exhibited sarcomeric gaps, compared with control (w^1118^>*ifc* RNAi ^KK^) hearts (Fig. 2G). This constricted heart phenotype is reminiscent of the KD of two other ceramide biosynthetic genes, *lace* and *schlank* (Walls et al., 2018). Importantly, cardiac-specific KD of *ifc* does not affect global TG levels, as observed in both *ifc^4^* mutants and global *ifc^4^* KDs (Walls et al., 2013). These cardiac effects correlated with a loss in cardiac *ifc* mRNA expression (Fig. S1) relative to UAS-*ifc* RNAi controls. The opposing phenotypes in heart function upon global versus heart-specific *ifc* loss-of-function suggests that the systemic effects of sphingosine Δ4 desaturase perturbation versus cardiac-specific perturbation differentially affect cardiac function. This observation might result from a variety of possible changes in either systemic and/or cardiac-specific sphingolipid, fatty acid and/or triglyceride metabolism.

### Mutant *ifc^4^* flies exhibit reduced *Sk1* expression

We previously reported that *ifc^4^* mutants exhibit reduced C_14:1_ ceramide levels, but accumulated larger amounts of C_14:0_ dihydroceramide and C_14:2_ ceramide dienes, with total ceramide levels in these flies slightly decreased relative to wild-type flies (∼10%) (Walls et al., 2013). Interestingly, we had previously observed that *Sk2* KD flies accumulate ceramide, dihydroceramide, sphingosine and dihydrosphingosine, but flies with KD of *Sk1* preferentially accumulate ceramide, ceramide dienes, sphingosine and sphingosine dienes. These results suggest that Sk1 and Sk2 exhibit differential activity regarding the metabolism of specific ceramide and sphingosine subspecies based on the degree of sphingoid base chain saturation. Hence, we sought to determine whether lipidomic profiles in *ifc^4^* mutants are accompanied by differential expression of genes involved in sphingolipid metabolism. We compared *lace*, *ifc*, *Sk1*, *Sphingosine kinase II* (*Sk2*) and *Sphingosine 1-phosphate lyase* (*Sply*) mRNA expression in *ifc^4^* mutants with wild-type controls (refer to fig. 1A in Walls et al., 2018).

**Fig. 3.**
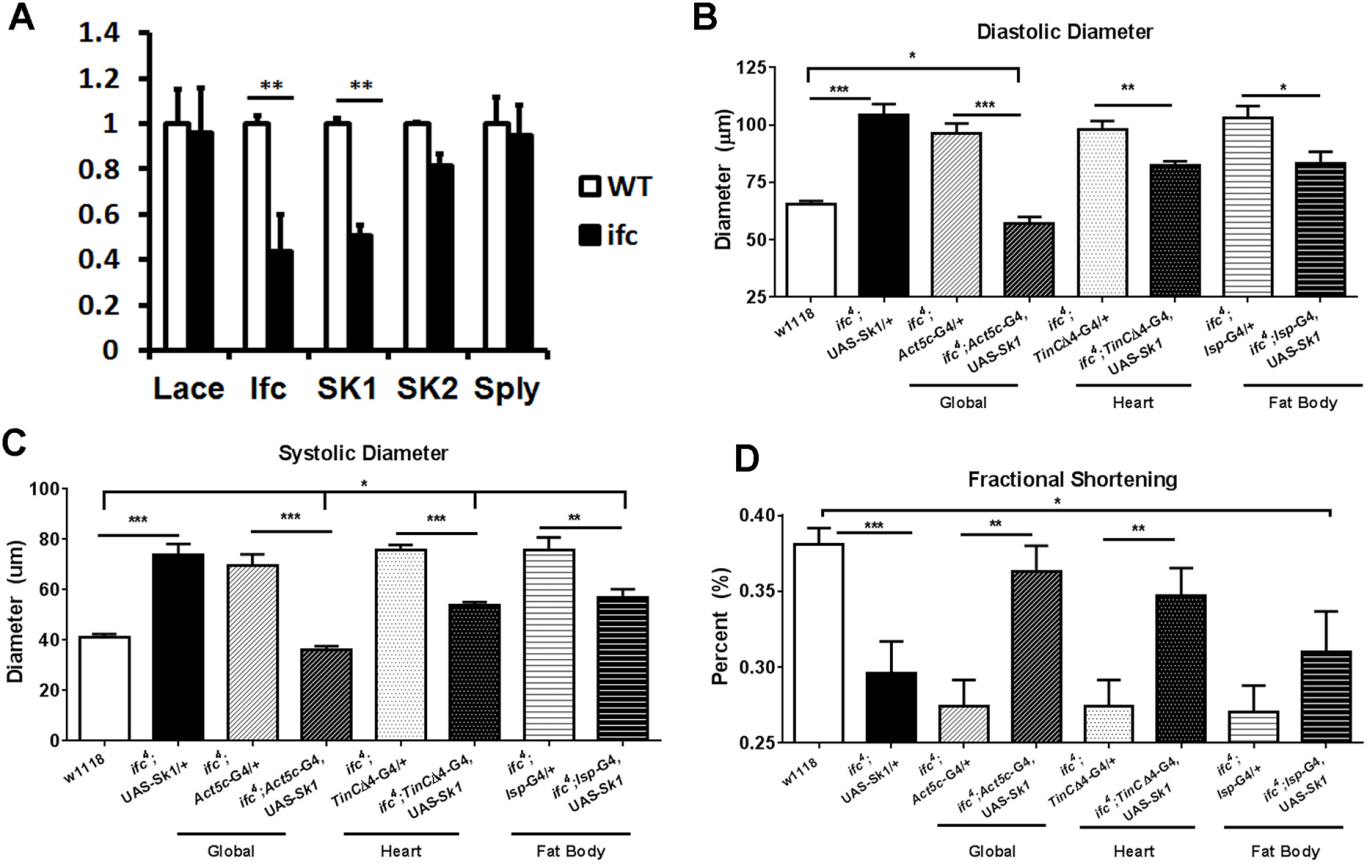
**The *ifc^4^* mutant cardiac phenotypes are Sk1 dependent.** (A) The *ifc^4^* mutants exhibit a reduction in both *ifc* and *Sk1* expression compared with Canton-S (wild-type) controls. (B-D) The *ifc^4^* mutants were compared with global (*ifc^4^*; Act5c-G4/UAS-*Sk1*), heart-specific (*ifc^4^*; Tinc4-G4/UAS-*Sk1*) or fat- body-specific (*ifc^4^*; *lsp*-G4/UAS-*Sk1*) *Sk1*-overexpressing lines in an *ifc^4^* mutant background. Diastolic diameter (B), systolic diameter (C) and fractional shortening (D) were assessed. Controls: *w1118*. Drivers: *ifc^4^*; *Act5c*-G4/+, *ifc^4^*; *tincΔ4*/+; *ifc^4^*; *lsp*-G4/+. UAS: (*ifc^4^*; UAS-*Sk1*/+). Multiple comparison two-tailed Student's *t*-test. *P<0.05; **P<0.01; ***P<0.001. Error bars=s.e.m. *n*=30 flies.

*ifc^4^* mutants exhibited a >65% global reduction in *ifc* expression (Fig. 3A). Interestingly, these flies also exhibit reduced *Sk1* expression (Fig. 3A). We previously reported that *ifc^4^* mutants exhibit an accumulation of C_14:2_ ceramide dienes (Walls et al., 2013) and that global *Sk1* KD flies also exhibit an accumulation of C_14:2_ ceramide dienes. Thus, the accumulation of ceramide dienes in *ifc* mutants might be due to concomitant reduction in *Sk1* expression. No change in the mRNA levels of *lace*, *Sk2* or *Sply* was observed (Fig. 3A).

### Inducing expression of *Sk1* reversed the cardiac dilation in *ifc^4^* mutants

Although *Sk1* is widely expressed throughout various fly tissues, it is not expressed in the heart (Chintapalli et al., 2007; Leader et al., 2018). Additionally, global but not heart-specific reduction in *Sk1* function causes cardiac dilation, which correlates with a respective loss of global *Sk1* expression (Walls et al., 2018). This observation suggests that the concomitant reduction of systemic *Sk1* in *ifc^4^* mutants might override the constriction phenotype observed in heart-specific *ifc* KD and lead to cardiac dilation (Fig. 1).

As *ifc^4^* mutants exhibited reduced expression of *Sk1*, we sought to determine whether expression of *Sk1* could rescue the dilation phenotype. Indeed, global overexpression of *Sk1* using a UAS-Sk1 construct (Herr et al., 2004) and global actin driver UAS-Sk2 in *ifc* mutants reversed the dilation and normalized contractility (Fig. 3B-D). Next, we ectopically expressed *Sk1* specifically in the heart of *ifc^4^* mutants to determine whether cardiac-specific expression of *Sk1* was sufficient to confer cardioprotection from the effects of systemic *ifc^4^* mutants. Cardiomyocyte-specific *Sk1* expression with the *TinCΔ4*-Gal4 driver (Lo and Frasch, 2001) in the *ifc^4^* mutant background partially reversed the dilation and contractility phenotype of these mutants (Fig. 3B-D).

As the fat body has a major role in global lipid homeostasis and performs roles comparable to both adipose tissue and liver in mammals (Banerjee et al., 2012), we utilized the fat-body-specific *lsp2*-Gal4 driver to determine whether *Sk1* overexpression in the fat body could also prevent the phenotype observed in *ifc^4^* mutants. Fat body expression of *Sk1* also partially rescued the dilation phenotype, but not the reduced fractional shortening of *ifc^4^* mutants. (Fig. 3B-D). Taken together, these data suggest that suppression of *Sk1* expression might underlie the lipotoxic cardiac phenotype observed in *ifc^4^* mutants.

### Preventing Akh loss in *ifc* mutants improves cardiac function

**Fig. 4.**
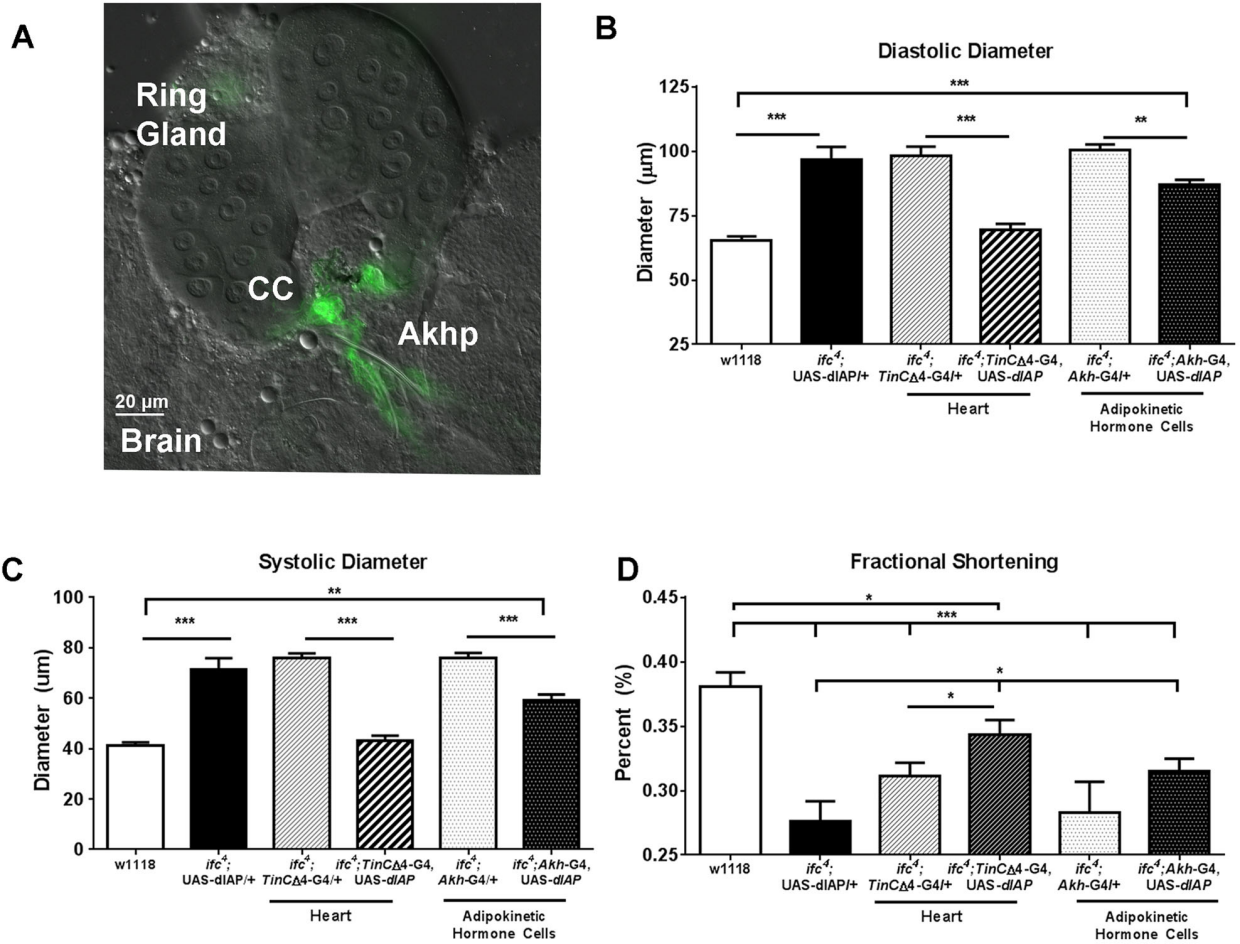
**The *ifc^4^* mutant cardiac phenotypes are dependent on Akhpcs and caspase.** (A) Wild-type 3rd instar larvae show specificity of Akh driver expression (*Akh*-G4; UAS-*GFP*), where Akhpcs are located next to the corpus cardiacum (CC) on the ring gland of the larval fly brain. (B-D) Cardiac-specific overexpression of caspase inhibitor *dIAP* is sufficient to normalize diastolic diameter (B) and systolic diameter (C), with a partial improvement in fractional shortening (D) in *ifc^4^* mutants. Akhpc rescue by *dIAP* overexpression partially reduced diastolic and systolic diameter and improved fractional shortening.

Akhpcs (Fig. 4A) have a glucagon/β-adrenergic-type role in regulating glycogen and TG storage in flies (Gronke et al., 2007). Akhpc-specific expression of *dIAP* in *ifc^4^* mutants reversed the loss of Akhpcs (Walls et al., 2013). Thus, we tested if Akhpc restoration also has a protective effect on cardiac function. Indeed, *ifc^4^*-induced dilation was partially reversed upon Akh cell restoration, but loss of fractional shortening was not significantly improved (Fig. 4B-D). The partial rescue is probably achieved through the restoration of Akhpc-mediated TG utilization, thus leading to a reduction in both systemic and heart TGs. However, this finding also suggests that systemic loss of sphingosine Δ4 desaturase activity might still have a role in the induction of moderate cardiac dilation and loss of shortening, despite preventing Akhpc ablation in *ifc^4^* mutants (see Walls et al., 2013 for dIAP-mediated prevention of Akhpc loss in *ifc^4^* mutants). Taken together, systemic factors involving *ifc^4^* loss of function, independent of Akhpc viability, contribute to the observed cardiac defects.

### Cardiac expression of *dIAP* is cardioprotective in *ifc^4^* mutants

Previously, we provided evidence that ceramide-associated lipotoxic cardiomyopathy was in part mediated by caspase activation through interactions with Annexin X (Walls et al., 2018). Furthermore, we observed that cardiac-specific KD of Annexin X (a caspase activator) or cardiac-specific overexpression of *dIAP* (a caspase inhibitor) protected the heart against ceramide-associated lipotoxicity. Importantly, flies with cardiac-specific expression of *dIAP* alone exhibit no cardiac phenotype (Walls et al., 2018). We sought to determine whether caspase activation specifically in the heart was important in the induction of the dilated lipotoxic cardiomyopathy observed in *ifc^4^* mutants. Hence, we expressed *dIAP* in *ifc^4^* mutant flies. Strikingly, cardiomyocyte-specific expression of *dIAP* exhibited a strong cardioprotective effect: both diastolic and systolic diameters were normalized in *ifc^4^* flies upon *dIAP* expression and fractional shortening increased, indicative of substantially improved cardiac function. These data suggest that cardiac-specific inhibition of caspase-activation through *dIAP* overexpression in *ifc^4^* mutants prevents the deleterious effects of mutation on cardiac function.

## DISCUSSION

Our results obtained using a unique combination of spatially controlled genetic interactions provide evidence that co-regulation of the genes *ifc* and *Sk1* can differentially affect cardiac function in a tissue-specific manner. Specifically, the *ifc^4^* mutation leads to systemic reduction in *Sk1* expression. We propose that this phenotype is associated with lipotoxicity generated in part by systemic changes in either sphingolipid, fatty acid and/or TG metabolism, which we have previously reported (Walls et al., 2013; Fyrst et al., 2009; Fyrst et al., 2008; Struckhoff et al., 2004). Additionally, global *ifc* KD adversely affects cardiac function with similar, albeit less severe, lipotoxic cardiomyopathy phenotypes. This finding is consistent with other ceramide-associated models of lipotoxic stress in the fly heart (Walls et al., 2018).

We also previously reported that global *Sk1* KD induced the accumulation of ceramide and sphingosine dienes (Walls et al., 2013), which correlated with a dilated lipotoxic cardiac phenotype in these flies (Walls et al., 2018). Cardiac-specific knockdown of *Sk1* produced no phenotype, which was expected as *Sk1* is not expressed in the heart (Walls et al., 2018). Here, we showed that in *ifc^4^* mutants global *Sk1* expression is suppressed, which might account in part for the observed dilated phenotype in these flies. Importantly, global ectopic expression of *Sk1* in an *ifc^4^* mutant background conferred a slightly restricted phenotype relative to control flies. Additionally, heart-specific ectopic expression of *Sk1* in *ifc^4^* mutants mitigates the dilated phenotype observed in *ifc^4^* mutants. Similar effects were observed when *Sk1* was ectopically overexpressed in the fat body. These data suggest that modulation of genes regulating both ceramide synthesis and degradation, specifically in the heart as well as the periphery, affect cardiac structure and function. Hemolymph-mediated transport of cardiotoxic sphingolipid metabolites between the fat body and heart might underlie these processes, although the effects of sphingosine Δ4 desaturase activity and its effects on general lipid metabolism are also likely to be major contributing factors (Matsuo et al., 2019). Interestingly, cardiac-specific *ifc* KD confers a restricted phenotype. This is consistent with previously reported models, where cardiac-specific KD of the ceramide biosynthesis genes *lace* and *schlank* also conferred a restricted phenotype (Walls et al., 2018). This outcome probably occurs through the heart autonomous reduction of ceramide production, without a concomitant reduction in global *Sk1* expression, and through changes in Sk1 target sphingolipid intermediates.

**Fig. 5.**
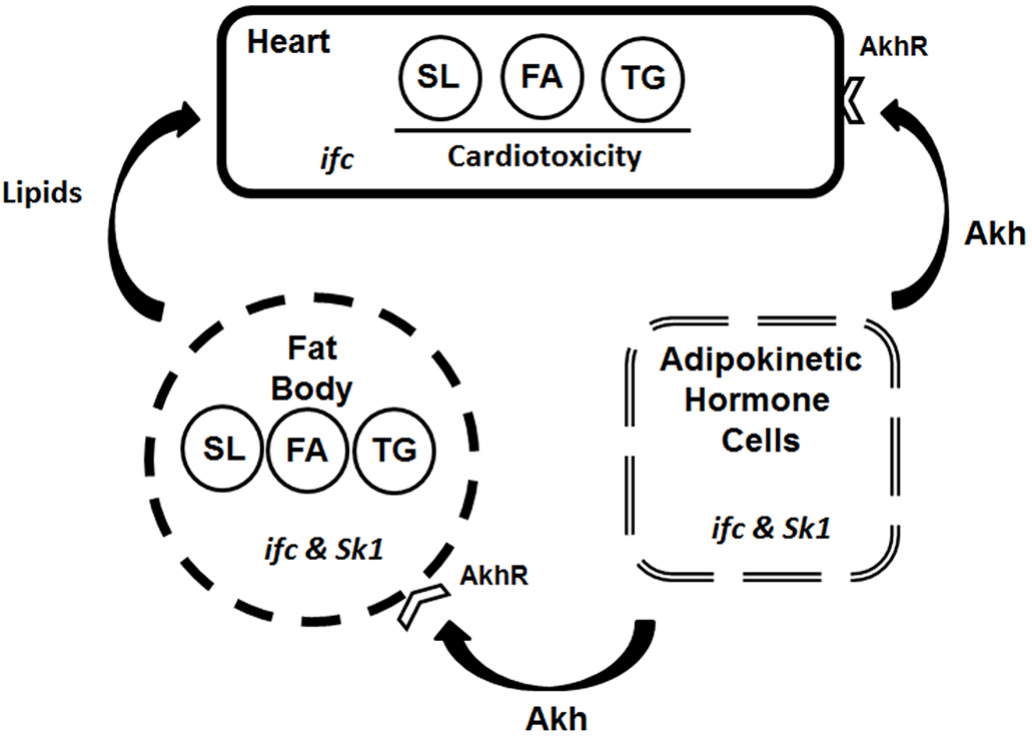
**Interplay between cardiac, fat body and Akhpc tissues.** Cardiac function in *Drosophila* is probably influenced by heart autonomous sphingolipid (SL), fatty acid (FA) and triglyceride (TG) pools, as well as by systemic pools in the fat body. The enzymatic functions of sphingosine Δ4 desaturase and sphingosine kinase 1 are essential for Akhpc viability, which is an effector of TG stores in both the fat body and the heart (Gronke et al., 2007). Modulation of the TG stores is also likely to influence SL and FA pools through shared metabolic pathways (TG→FA→SL biosynthesis; FA+diacylglycerol→TG). This interconnection is functionally analogous to the interplay of mammalian organs of the heart, liver/adipose tissue and pancreas (α-cells)/adrenal gland in mammals (Gronke et al., 2007).

However, it has yet to be determined directly whether the observed cardiac dysfunction in *ifc^4^* mutants is associated with cardiac accumulation of ceramide and sphingolipid species that originated in the periphery (fat body, etc.). The development of reliable analytical methods for measuring sphingolipidomes in the fruit fly heart will be needed to determine whether these cardiotoxic sphingolipids are changed in the heart when modulated systemically, although our genetic data suggest that this is probably the case, together with probable changes in other lipotoxic lipid metabolites including TG. Nevertheless, tissue dysfunction, in either the fat body or Akhpc, is also likely to contribute to cardiac dysfunction when improperly regulated. Collectively, our data show that an important interplay exists between the heart, fat body and Akhpcs in regulating cardiac function under conditions of lipotoxicity in the fruit fly (Fig. 5). Similar observations have been made in mammalian models, in which interplay occurs between the heart, liver, adipose tissue and neuroendocrine regulatory organs.

## Supplementary Material

Supplementary information
