# Supplementary material for: Systemic and heart autonomous effects of sphingosine Δ4 desaturase deficiency in lipotoxic cardiac pathophysiology
Source: Dis Model Mech. 2020 Aug 14;13(8):dmm043083. doi: 10.1242/dmm.043083 (PMC7438009; doi:10.1242/dmm.043083)
Supplement: Supplementary information [file dmm-13-043083-s1.pdf]

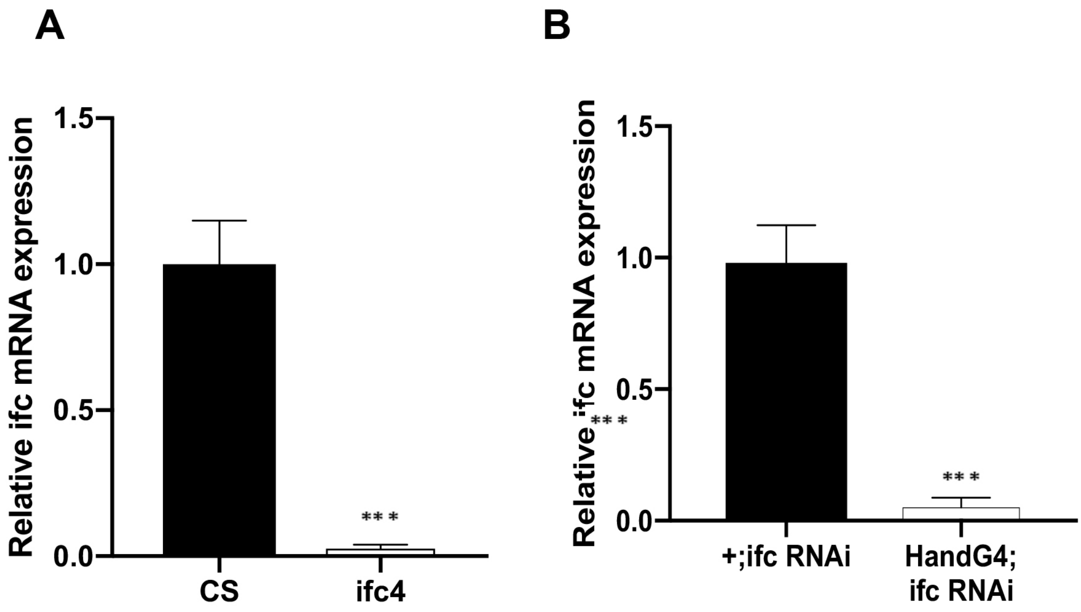

Fig. S1. qPCR Validation of Cardiac *ifc* expression. A) Cardiac expression of ifc mRNA in control (CD) versus ifc4 mutant. B) Cardiac expression of +;ifc RNAi controls (w1118 crossed into UAS-ifc RNAi) versus cardiac specific ifc RNAi knockdowns (HandG4 crossed into UAS-ifc RNAi).

Disease Models & Mechanisms • Supplementary information
